# Complications in silane-assisted GaN nanowire growth†

**DOI:** 10.1039/d2na00939k

**Published:** 2023-04-20

**Authors:** Nian Jiang, Saptarsi Ghosh, Martin Frentrup, Simon M. Fairclough, Kagiso Loeto, Gunnar Kusch, Rachel A. Oliver, Hannah J. Joyce

**Affiliations:** a Department of Engineering, University of Cambridge 9 JJ Thomson Ave Cambridge CB3 0FA UK nj279@cam.ac.uk; b Department of Materials Science and Metallurgy, University of Cambridge 27 Charles Babbage Rd Cambridge CB3 0FS UK

## Abstract

Understanding the growth mechanisms of III-nitride nanowires is of great importance to realise their full potential. We present a systematic study of silane-assisted GaN nanowire growth on *c*-sapphire substrates by investigating the surface evolution of the sapphire substrates during the high temperature annealing, nitridation and nucleation steps, and the growth of GaN nanowires. The nucleation step – which transforms the AlN layer formed during the nitridation step to AlGaN – is critical for subsequent silane-assisted GaN nanowire growth. Both Ga-polar and N-polar GaN nanowires were grown with N-polar nanowires growing much faster than the Ga-polar nanowires. On the top surface of the N-polar GaN nanowires protuberance structures were found, which relates to the presence of Ga-polar domains within the nanowires. Detailed morphology studies revealed ring-like features concentric with the protuberance structures, indicating energetically favourable nucleation sites at inversion domain boundaries. Cathodoluminescence studies showed quenching of emission intensity at the protuberance structures, but the impact is limited to the protuberance structure area only and does not extend to the surrounding areas. Hence it should minimally affect the performance of devices whose functions are based on radial heterostructures, suggesting that radial heterostructures remain a promising device structure.

## Abstract 2

Understanding the growth mechanisms of III-nitride nanowires is of great importance to realise their full potential.

## Graphical abstract

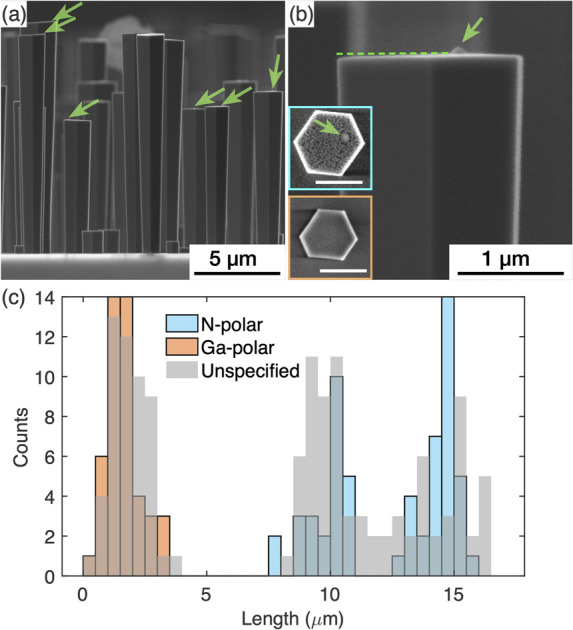

## 1. Introduction

III-nitride nanowires have attracted research interest from both academia and industry for their applications in light emitting diodes (LED) emitting in the deep UV and green to red ranges as they offer promising solutions for strain and point defect management.^1–4^ Their small footprint and quasi-1D geometry enables strain to relax elastically more efficiently than their planar counterparts, and thus reduces the formation of misfit dislocations.^5,6^ As a result, III-nitride nanowires can have higher charge carrier lifetimes and electron mobilities compared to their bulk counterparts.^7^ III-nitride nanowires are also considered as potential candidates for photoelectrochemical (PEC) water-splitting applications.^8–12^ The nanowire geometry brings large surface area-to-volume ratios which enables efficient charge transfer across interfaces. Furthermore, nanowire arrays exemplify non-planar absorber geometries that can improve light extraction and absorption.^13^

Amongst the fabrication methods for group-III-nitride nanowires, self-assembled silane-assisted GaN growth (referred to as silane-assisted growth) *via* metal organic chemical vapour deposition (MOCVD) offers a promising opportunity for mass production of GaN nanowires with a fast growth rate along the *c*-axis. First demonstrated by Koester *et al.* in 2010,^14^ this method typically involves the treatment of the sapphire substrate in an NH_3_ atmosphere (nitridation) followed by a short GaN seed growth step (nucleation step) to provide GaN nuclei for subsequent nanowire growth. Silane (SiH_4_) is then introduced to increase the growth rate along the *c*-direction and to suppress lateral growth.^14–16^ This method requires no *ex situ* surface preparation (*e.g.* masks for selective area growth) of the substrate and no extrinsic catalyst, unlike other nanowire growth methods.^17^ This offers a way to fabricate nanowire structures without introducing potential contamination to the material and reactors, which is important for achieving compatibility with industrial manufacturing routes. Without the need of a dielectric mask, it also offers a lithography-free means to achieve nanowires.

The silane-assisted nanowire growth method has the following features. First, it offers growth rates from a few tens of micrometres per hour to greater than 100 μm h^−1^ on the nanowire axis along the 〈0001〉 direction.^14,16^ The enhancement of the growth rate is predominately observed for the N-polar -*c* (0001̄) plane, although nanowires with N-polarity, Ga-polarity and mixed polarities have been observed in the literature.^16,18,19^ Second, GaN nanowires grown by this method often exhibit a simultaneously formed Si-rich layer on the sidewall facets^15,20–22^ This Si-rich layer was reported to be responsible for the growth rate enhancement by preventing further deposition of GaN on the sidewall facets.^20^ Although this Si-rich layer deteriorates the quality of the InGaN shell layers grown outside of it,^21–23^ its impact can be reduced and high quality core–shell structures can be achieved by optimising the structures^21,22^ or removing the Si-rich layer prior to the growth of InGaN shell layers.^23^

Despite the wide adoption of this method, questions remain about the growth mechanism. Tessarek *et al.*, attributed the fast growth rate to the passivating Si-rich layers on the sidewall facets, which increases the amount of Ga adatoms arriving at the growth front at the top surface, thus enhancing the growth rate for the -*c* (0001̄) plane.^20^ This indicates that the growth is a mass flow-controlled process, and the growth rate should be faster for nanowires with smaller diameters.^24^ Yet, no diameter dependence of the growth rate was observed.^25^ Tessarek *et al.* also found that (0001) Ga-polar planes have a faster growth rate than (0001̄) N-polar planes, forming a protuberance structure on top of a Ga-polar domain core in the nanowire surrounded by a N-polar shell.^16^ This is in contrast to other literature on the growth of GaN nanowires with mixed-polarity, where Ga-polar domains were either shorter or the same height as the N-polar domains adjacent to them.^18,19,25^ In addition, although silane was reported to enhance the growth on both the N- and Ga- polar planes, the growth enhancement on Ga-polar planes was not as strong as that on the N-polar planes.^15,16^ These phenomena suggest that extra mechanisms were involved in silane-assisted growth. In addition, the nucleation step, performed prior to silane introduction, has received comparatively little attention, yet is crucial for controlled nanowire growth.

This paper examines the role of the nucleation step and the subsequent growth mechanism of silane-assisted GaN nanowires. We studied the evolution of the sapphire substrate surface prior to the silane-assisted nanowire growth step, revealing the importance of surface modification changing AlN to Al(Ga)N during the nucleation step. Length statistics of the grown GaN nanowires showed that the growth rates along the 〈0001〉 *c*-axis of Ga-polar nanowires were much slower than those of the N-polar nanowires. Protuberance structures were found on the top surface of the N-polar nanowires and these are related to Ga-polar domains within the nanowires. We proposed that the inversion domain boundaries of these mixed domains within the same nanowire act as energetically preferred nucleation sites by which they increase the nucleation rates and contribute to the fast growth rate on the -*c* plane.

## 2. Experiment

### 2.1 MOCVD growth

Self-assembled GaN nanowires were grown on *c*-plane sapphire substrates (α-Al_2_O_3_, with 0.25° miscut towards the [112̄0] *a*-direction) in a Veeco Propel™ PowerGaN MOCVD reactor, using ammonia (NH_3_), trimethylgallium (TMGa) and silane (SiH_4_) as precursors and hydrogen (H_2_) as the carrier gas. The susceptor temperature and wafer surface reflectivity during growth were monitored in real time by a RealTemp 200® module installed within the reactor. The sapphire substrates were loaded into the reactor without any pre-treatments. Once loaded the substrate surfaces were first cleaned *in situ* in a H_2_ atmosphere at 1060 °C for 5 min (referred to as the high temperature annealing step), followed by a 10 min nitridation treatment at 1080 °C in NH_3_ with a flow rate of 15 slm. The long nitridation at high temperatures is reported to eliminate mixed polarity and promote pure N-polar growth.^25^ After nitridation, the reactor temperature and NH_3_ flow rate were then decreased to prepare for the GaN nanowire growth.^14,25^ A nucleation step was adopted to initiate the GaN nanowire growth by switching on TMGa with a flow rate of 744 μmol min^−1^ and maintaining a [NH_3_]/[TMGa] ratio of 12. After the nucleation step TMGa was switched off for 15 s (referred to as a growth interruption) to adjust the flow rate of the precursors for the subsequent silane-assisted growth at a [NH_3_]/[TMGa] ratio of 6 and a [SiH_4_] flow of 0.8 μmol min^−1^ for 10 min. Detailed precursor profiles during the growth are shown in ESI (Fig. S1).†

To study the impact of the nucleation step on the nanowire growth, a set of GaN samples (Set A) was grown with and without a nucleation step. For the growth without the nucleation step, silane-assisted growth was carried out straight after ramping the growth parameters from the nitridation conditions to the nanowire growth conditions. To study the morphology evolution by the individual growth steps, an additional set of three samples (Set B) was prepared, for which the growths were terminated after the high temperature annealing step, nitridation step and nucleation step (5 s) respectively. For the sample terminated after the nucleation step, the sample was cooled down immediately to 750 °C within 4 min in a N_2_ atmosphere and with a NH_3_ flow rate of 8.93 mmol min^−1^ (as used for the nucleation step) to preserve the surface structures.

### 2.2 Characterisation

The morphology of the GaN nanowires was studied by secondary electron (SE) imaging in a FEI Helios dual beam focused ion beam/scanning electron microscope (FIB/SEM). The polarity of the GaN nanowires was probed by treating them with 25% (weight) KOH solution at room temperature for 30 min.^19,26^ Due to different etching rates of Ga- and N-polar surfaces, the Ga-polar surfaces showed no change in morphology while the N-polar surfaces are etched.^19^ Atomic force microscopy (AFM) with a Bruker Dimension Icon Pro in Peakforce Tapping® mode with Scanasyst-Air probes were used to study the surface morphologies of the samples in Set B and the top surfaces of the GaN nanowires. X-ray photoelectron spectroscopy (XPS) was used to study the surface chemistries of the samples in Set B. These samples were stored under vacuum to reduce post-growth contamination prior to the XPS analysis. XPS analysis was carried out using an Escalab 250XI spectrometer from Thermo Fisher Scientific, operating in constant analyser energy mode. The energetic position of the C 1s emission line at a binding energy of 284.8 eV was chosen to calibrate the energy scale of the spectra.^27^ (Scanning) Transmission electron microscopy (STEM/TEM, FEI Tecnai Osiris FEG-TEM) running at 200 kV and beam current of 100 pA was used to study the crystal structures of the grown GaN nanowires. Energy dispersive X-ray (EDX) analysis was performed with SuperX detector and convergent beam electron diffraction patterns (CBED) were recorded using Gatan OneView camera. To analyse the optical emission properties of the samples, continuous-wave cathodoluminescence (CL) was performed using an Attolight Allalin 4027 Chronos CL SEM at room temperature with an acceleration voltage of 6 kV and a beam current of 5 nA.

## 3. Results & discussion

### 3.1 Essential nucleation step

**Fig. 1:**
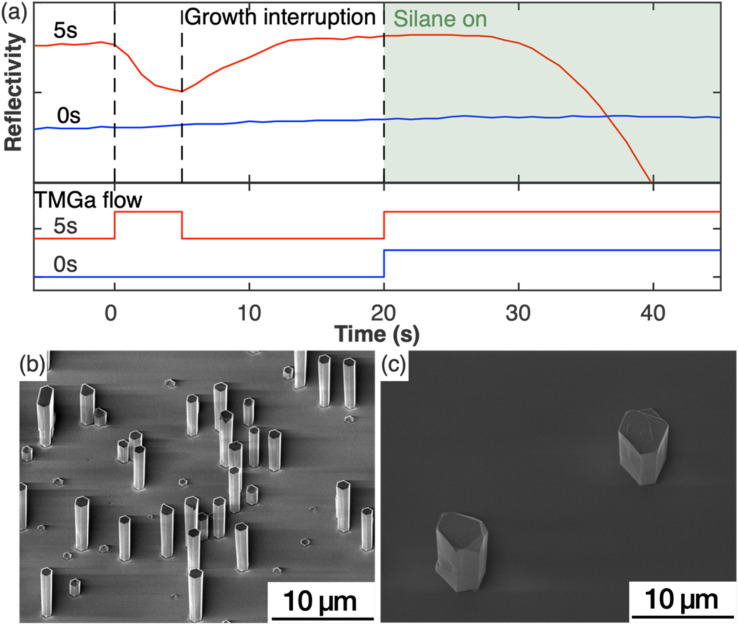
(a) *In situ* reflectivity transients and the TMGa flow during the growth with (red) and without (blue) nucleation step. The shaded box highlights the introduction of silane flow. (b and c) SE images of GaN nanowires grown with (5 s) and without (0 s) nucleation step.

To study the impact of the nucleation step on the nanowire growth, two samples were grown under the same growth parameters except for the nucleation step (Set A). For one sample, this nucleation step was included in the growth, while it was left out for the second sample. Fig. 1(a) shows the *in situ* reflectivity signal measured during the growth run with the status of TMGa flow shown directly below. NH_3_ was provided through the whole process. For the sample with the nucleation step (red curve), TMGa was introduced to the reactor for 5 s. The reflectivity started dropping immediately as TMGa reached the reactor, indicating an increase in the surface roughness, possibly by the formation of 3D islands. As soon as the TMGa flow was stopped, the reflectivity stopped dropping and began to rise, indicating a reduction of surface roughness. This could be caused by the evaporation of the just formed 3D islands and/or rearrangement of the surface atoms into a flatter layer. The reflectivity recovered to a level similar to that before the nucleation step during the growth interruption. After both TMGa and SiH_4_ were switched on for the silane-assisted growth, the reflectivity stayed nearly constant for the first few seconds and then dropped. In comparison, no significant changes in the reflectivity data were observed during the whole growth process for the sample grown without the nucleation step (blue curve in Fig. 1(a)). This indicates that the surface remains relatively smooth and that the density of structures, which are newly formed during the silane-assisted growth phase and might be expected to cause significant roughening, is significantly lower compared to the sample grown with nucleation step.

These observations were confirmed by SEM measurements. Fig. 1(b and c) show the SE images of the samples grown with and without the nucleation step, respectively. The sample grown with nucleation step (5 s) (Fig. 1(b)) shows nanowire structures with diameters of (1.0 ± 0.1) μm and a height to width aspect ratio of ∼5 as measured from more than 50 nanowires. The density of the GaN nanowires is ∼10^5^ cm^−2^ measured from a total area of 7 × 10^−3^ mm^2^. All nanowires have smooth sidewalls despite variations in lengths. In comparison, the sample without a nucleation step (Fig. 1(c)), shows only sparsely scattered hexagonal pillars with an aspect ratio ∼1 and a density of less than 10^3^ cm^−2^ measured from an area of 0.8 mm^2^. Macroscale terraces were observed from the SE images on both the top surface and the sidewall facets of the hexagonal pillars. The differences between these two samples indicate that the nucleation step has modified the substrate surface in a way that is critical to encourage the growth of the GaN nanowires.

To understand the impact of the nucleation step on the subsequent silane-assisted nanowire growth, the surface evolution prior to the silane assisted growth step was investigated. The morphology and chemical composition of the samples in Set B, whose growths were terminated after the high temperature annealing, nitridation and nucleation steps respectively, were studied by AFM and XPS. The surface roughness of each sample is estimated from the root mean square (rms) roughness measured by AFM on two to three 0.5 × 0.5 μm^2^ areas. The resulting standard deviation is within the system resolution of 0.03 nm. For the XPS data, only the spectra for the elements that mark the surface changes are shown here, with the spectra of other elements shown in the ESI (Fig. S2–S5).†

**Fig. 2:**
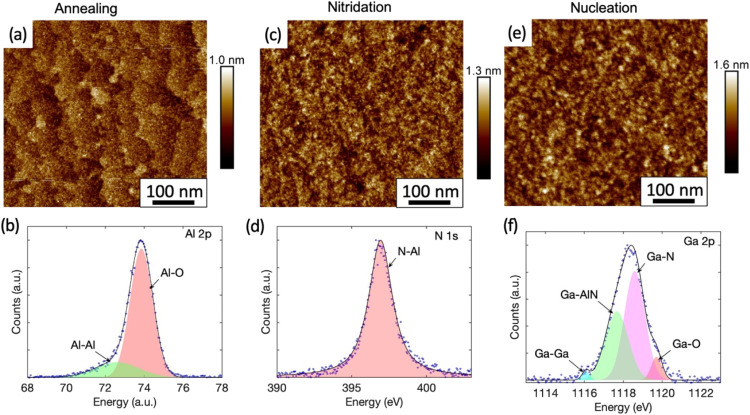
The AFM image (a) and the XPS spectra of Al 2p (b) from the sample with growth terminated after high temperature annealing. (c) And (d) show the AFM image and the XPS spectra of N 1s from the sample with growth terminated after nitridation step, respectively. (e) And (f) show the AFM image and the XPS spectra of Ga 2p from the sample with growth terminated after the nucleation step.

Fig. 2(a) shows the AFM image of the sample whose growth was terminated after the high temperature annealing. The atomic steps and terraces of the sapphire substrate are clearly visible with an rms roughness of 0.14 nm only. The chemical bonding analysis by XPS of the Al 2p peak is shown in Fig. 2(b). The measured data is best represented by fitting it with two Pseudo-Voigt distributions, with the dominant component at 73.89 eV representing Al–O bonds and another component at 72.60 eV representing Al–Al bonds.^28^ While Al–O bonding is expected for sapphire, the presence of Al–Al bonds is consistent with the formation of an Al-rich surface due to oxygen desorption during annealing in H_2_.^29,30^

The AFM and XPS analyses for the sample, which underwent a nitridation step after the high temperature annealing, are shown in the Fig. 2(c) and (d) respectively. The previously observed monolayer steps and terraces are no longer present in the AFM image (Fig. 2(c)) after nitridation and the rms surface roughness was increased slightly to 0.18 nm. Fine island structures were observed and particle size analysis show that they are within an average diameter of (7 ± 2) nm and height of (0.5 ± 0.1) nm. Protrusions related to the stress-enhanced material migration caused by compressive strain as seen in other work^31^ were not observed in our samples. The XPS analysis here revealed the appearance of a N 1s peak (Fig. 2(d)), which has not been observed for the as-annealed sample (Fig. S3†). The N 1s peak is attributed to N–Al bonds with a binding energy of 396.93 eV fitted by a Pseudo-Voigt peak. This is consistent with the formation of a widely reported thin AlN layer during the nitridation process, whose thickness typically ranges from a few Å to a few nm.^31,32^ However, the N–Al binding energy measured here is slightly lower than the reported value of (397.4 ± 0.3) eV.^33^ The peak shift might be caused by a high density of Al vacancies in the layer.^34^ Interestingly, the N–O component with a binding energy of (398.5–399.9 eV)^33,35^ expected for AlNO_*x*_ complexes is absent, indicating that the surface is fully covered by an AlN layer.

In short, the results show that during the nitridation step, N adatoms first substitute the remaining surface oxygen of sapphire,^36^ or bond with surface Al atoms directly,^37^ forming a N-terminated surface. Nitrogen adatoms then diffuse below the sapphire surface. There they continue replacing subsurface oxygen and form a thin, ill-defined AlN layer that possibly contains Al vacancies and substitutional O atoms most likely on N positions.^34,38,39^ The diffusion process and the drawing of oxygen out of the sapphire lattice are aided by the high temperature (1080 °C) and a long nitridation time (10 min). These nitrogen-rich conditions drive the formation of an N-polar surface compared to metal-polar III-nitride growth.^37^ The surface AlN layer has its 〈11̄00〉 in-plane direction aligned with the 〈112̄0〉 direction of *c*-plane sapphire.^37^ With the theoretical lattice mismatch of about 13.3% between both materials the critical thickness of AlN on sapphire is calculated to be less than one monolayer.^40^ As a consequence, parts of the AlN layer near the top surface are fully relaxed while for parts of the AlN formed sub-surface, the relaxation degree is limited by the sapphire matrix. As a result, the atomic steps observed after sapphire annealing disappeared from the substrate surface during the nitridation process, and a slight increase in surface roughness was observed.

The nitridation step is followed by the nucleation step. For the sample whose growth was terminated after the nucleation step, NH_3_ in the carrier gas of N_2_ was used after switching off TMGa to preserve the surface. For this sample, we have shown earlier (Fig. 1(a)) that the *in situ* reflectivity transient dropped as expected during the nucleation step. However, it started to increase and recovered to the level similar to that before the nucleation step. Therefore, we expect the surface of the sample is similar to the surface prior to the silane-assisted growth. This is confirmed by the AFM measurements in Fig. 2(e), where no obvious surface morphology change was observed in the sample compared to the sample which underwent the nitridation step only (Fig. 2(c)), except of a slight increase in the rms surface roughness to 0.24 nm. GaN islands with hexagonal shape were not observed in either AFM or SEM images, unlike previous reports.^14,16^ Only fine island structures, similar to those found on the sample that underwent the nitridation step only, were found. Particle size analyses show they are with an average diameter of (9 ± 4) nm and height of (0.7 ± 0.1) nm.

For the sample that underwent a nucleation step after nitridation, the XPS studies of the surface reveal a strong Ga 2p peak at 1118 eV (Fig. 2(f)). The best fit is given when this peak is deconvolved into four components as labelled. The major components are attributed to Ga–N bonds at 1118.59 eV (ref. 41) and Ga-AlN bonds at 1117.67 eV.^42^ A high energy peak at 1119.71 eV (ref. 43) is linked to Ga–O complexes. The presence of Ga–O complexes is likely attributable to oxidation *via* exposure to air over an approximately 2 hours period during sample transportation between the growth chamber and the XPS system. A small component from metallic Ga occurs in the XPS signal at a lower binding energy of 1116.08 eV.^43^ The XPS analysis suggests that some Ga may have diffused into the AlN layer previously created during the nitridation step to form an AlGaN alloy. This is consistent with a recent study by Liu *et al.*, showing that Al vacancies near the surface of the AlN formed during nitridation step will be occupied by upcoming metal atoms in the subsequent growth process.^44^

The *in situ* reflectivity transient, AFM and XPS analyses of the substrate surface combined with the growths with and without the nucleation step indicate that the surface modification that occurred during the nucleation step played a critical role in the resulting nanowire density and morphology. Hexagonal GaN islands with diameters of 50 nm to 150 nm that formed during the nucleation step – in the absence of silane – were reported to be critical to the morphology and crystal quality of the subsequent grown GaN nanowires.^14,16^ However, our results (*e.g.* in Fig. 1(b) and 2(e)) have shown that such large hexagonal GaN islands are not essential to achieve GaN nanowires. As shown in Fig. 1(a), the *in situ* reflectivity recovers to its full value within 7 seconds of the growth interruption, then plateaus at the same maximum value as seen for the original planar substrate. This observation indicates that the processes of evaporation of the newly-formed 3D GaN islands and/or rearrangement of the surface atoms into a flatter layer has occurred within this 7 seconds time period. In agreement, SEM and AFM images (Fig. 2(e)) show a surface devoid of hexagonal structures. We therefore choose to introduce a growth interruption of twice this duration (15 s) to ensure all islands are removed. Despite the removal of large hexagonal islands in the sample, successful growth of nanowires was achieved (Fig. 1(b)) evidencing our conclusion that large hexagonal islands are not the crucial element of the nucleation step in promoting nanowire growth. This is also supported by additional experiments in which we have grown nanowires in the same way with the only difference being the growth interruption between the nucleation step and the silane-assisted nanowire growth step (Fig. S1†). Although small local differences in the distribution and size of the nanowires can be observed (like in Fig. S6†), a statistical analysis across an area of about 1 cm^2^ per sample revealed that in both growths – with and without the growth interruption – nanowires with similar density and morphology have been formed. Thus, we conclude that it is the AlGaN transformed from the AlN layer instead of the hexagonal GaN islands that is critical for the subsequent silane-assisted GaN nanowire growth. This means it may not be appropriate to correlate the GaN islands formed during the nucleation step with the final GaN nanowires in literature^14,16^ where very different correlations between the nucleation time and the resultant nanowire morphologies and densities were reported.

### 3.2 Ga- and N-polar GaN nanowires

**Fig. 3:**
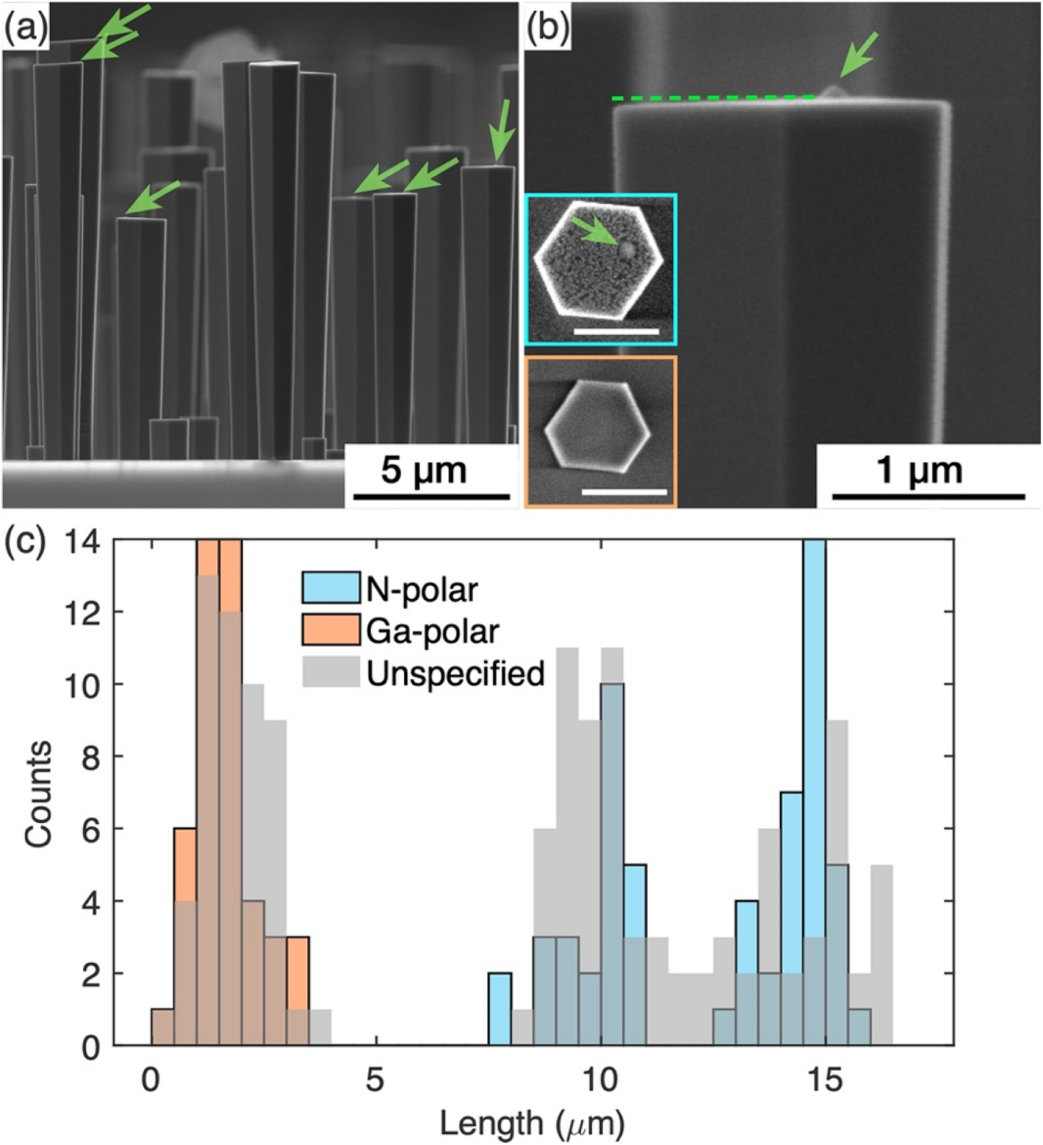
(a) SE images of GaN nanowires on sapphire substrate. A protuberance structure is visible in most of the tall nanowires, as pointed out by the green arrows. (b) Shows a zoomed in image of the protuberance structure. The dashed line represents the projection of the 〈0001〉 plane. Insets in (b) show the top surface morphology after KOH etching for the tall and short nanowire, respectively. The scale bars in the insets are 1 μm. (c) Nanowire length distribution. The histogram in grey was measured prior to the KOH etch test. The orange and blue histograms were measured after the KOH etch test, which discriminated the Ga- and N-polar nanowires.

Further detailed study was carried out on the GaN nanowires grown with the nucleation step to understand the impact of the surface modification that occurred during the nucleation step, on the subsequent silane assisted growth. AFM was used to image the topography of the growth front, *i.e*. the top surface of the GaN nanowires. For these studies, GaN nanowires were grown with double the time of the silane-assisted growth step as the nanowires shown in Fig. 1(b) to achieve larger diameters to facilitate AFM scans later. To assess and compare the overall quality of the nanowires, SE images as shown in Fig. 3(a) have been collected. These images show that all nanowires have a relatively flat top surface but, in some cases, a protuberance structure can be resolved on the top surface, as indicated by the green arrows in Fig. 3(a). Fig. 3(b) shows a zoomed-in picture of a nanowire top end with the protuberance structure close to one side. The projection of the -*c*-plane (perpendicular to the ridge in the middle where two sidewall facets meet) is indicated by the dashed line. We note that the top surface is tilted slightly with areas near the protuberance structure higher than the areas further away.

The polarity of the nanowires was identified from the different etch behaviours of the surfaces during KOH treatment. It is widely known that N-polar surfaces are etched by KOH more rapidly than Ga-polar surfaces.^19,26^ Typical top surface profiles of N- and Ga-polar surfaces after KOH treatment are shown as insets in Fig. 3(b). For Ga-polar nanowires (orange box) the surface remains relatively smooth after KOH etching, while the surface of N-polar nanowires (blue box) shows a significant roughening due to the etching process. Interestingly all nanowires with a protuberance structure on the top surface are N-polar. In these nanowires, the protuberance structure stood out on the top surface when the surrounding area turned rough due to the KOH etching, as shown in the SE image (Fig. 3(b)). Due to the tilted surfaces, the polarity of the protuberance structures cannot be identified by KOH etching.^18^ Similar protuberance structures have been reported in literature and their polarities were identified as Ga-polar by TEM.^16^

The presence of both N-polar and Ga-polar nanowires co-existing on the same sample is consistent with previous reports.^25^ It has been suggested that Ga-polar nanowires and domains form as a result of incomplete nitridation of the sapphire surface.^25^ However, our XPS studies show that the sapphire substrates were completely covered with a thin AlN layer after the nitridation step. The fact that both Ga- and N-polar GaN nanowires grew simultaneously in our samples suggests a different origin of the growth on Ga-polar planes. Indeed, molecular dynamic simulations of GaN nanorods showed that inversion domains can form as a result of the surface tension relaxation, the impact of which decreases as the diameter and length of the nanorod increases.^45^ We thus suggest that the Ga-polar domains in our GaN nanowires originated from the Al(Ga)N layer as a result of both the strain and surface tension relaxation. The same mechanism could explain the origin of the fully Ga-polar nanowires that we observe co-existing with N-polar nanowires.

Statistics of the nanowire lengths measured from over 100 nanowires are shown in Fig. 3(c). The nanowires fall into two categories – the ones longer than 8 μm and the ones shorter than 4 μm. The length distribution was superimposed with the length measured from the nanowires treated with KOH, where the lengths of N-polar and Ga-polar nanowires were measured separately. Note that the etch conditions used were sufficient to roughen the N-polar surfaces, but not sufficient to shorten the nanowires significantly. Therefore, the pre- and post-etch distributions are similar. The results reveal that the length distributions of Ga- and N-polar nanowires overlap with the shorter and longer ones, respectively. Thus, we conclude that the shorter nanowires are Ga-polar and the longer ones are N-polar. The protuberance structures and the tilt of the top surface were commonly observed in the N-polar/longer nanowires, while neither protuberance structures nor the tilt of the top surface were seen in the Ga-polar/shorter nanowires. In the histogram in Fig. 3(c), two clearly separated Gaussian distributions of the lengths of the N-polar GaN nanowires can be identified centred at 9.5 and 14.5 μm. The mechanisms leading to these two different distributions will be discussed in future work.

In our study, both Ga-polar and N-polar nanowires exhibited relatively flat, *c*-plane-like top facets, notwithstanding the protuberance structure and slight incline of the top facet of the N-polar nanowires. Flat *c*-polar top facets are commonly reported for N-polar nanowires.^14,16,19,25^ Ga-polar nanowires have been reported with pyramidal (non-*c*-plane) top facets^46^ and flat *c*-plane top facets similar to our observations.^25^ It is worth noting that the faceting behaviour is known to be sensitive to the growth method (*e.g.* selective area growth, vapour–liquid–solid or silane-assisted) and the growth parameters, particularly the V/III ratio.^25,46^

### 3.3 Nucleation surrounding the protuberance structure

The appearance of protuberance structures on top of N-polar nanowires raises questions regarding their origin. As the nanowires were grown under Ga-rich conditions, one option might be that the protuberances result from metal Ga droplets present during growth. No such droplets were observed in our post-growth *ex situ* measurements, but their absence does not conclusively rule out the possibility of Ga droplet-mediated growth, because Ga droplets may react with ammonia to form GaN during the cool-down to room temperature. Nevertheless, only N-polar nanowires feature protuberances and not Ga-polar nanowires, whereas metal droplets, if responsible for the protuberance, would likely affect both N-polar and Ga-polar wires. To explore the origin further, we now explore the topographic and structural properties of the N-polar nanowires.

**Fig. 4:**
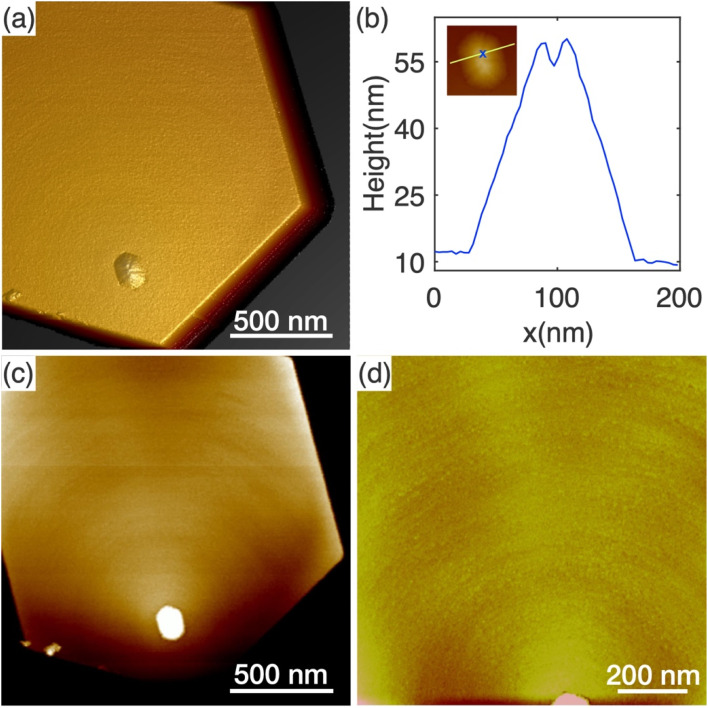
AFM images of the top surface of a typical tall nanowire. (a) Height error image showing qualitative changes in the topography. (b) Height line profile across the protuberance structure, showing a trench on the surface. The inset AFM image shows the line and the position of the trench. (c and d) Reveal the rings co-centered at the protuberance structure.

The AFM topography of the top surface of a typical N-polar long nanowire is shown in Fig. 4. Fig. 4(a) shows a 3D height error image where a pyramid-like protuberance is clearly visible towards one corner on the top surface. The base shape of the protuberance structure appears to be roughly hexagonal with boundaries parallel to the nanowire sidewalls. A few trench-like features were noticeable on the surface of the protuberance structure. A height line profile across the protuberance structure reveals a trench with a depth of 5.6 nm and width of 14.2 nm (Fig. 4(b)). Similar structures were found on the top of all four nanowires studied by AFM, as shown in Fig. S7.†

Height analysis of the flat area of the top surface reveals a series of concentric monolayer height features around the protuberance structures, as shown in Fig. 4(c). This characteristic is confirmed and highlighted in Fig. 4(d) with the zoomed-in AFM image of only the flat area. Different from the atomic steps observed in typical spiral growth,^47^ the rings are much denser with much smaller distances in between. As a result, it is rather challenging to determine the nature of the features between spirals and rings. Nonetheless, these features indicate that the edges of the protuberance structures were preferred nucleation sites for new layers of GaN. This resulted in the slight tilting of the top surface observed in Fig. 3(b). However, this tilt is not large enough to form a new crystallographic plane.

The concentric features at the top surface of nanowires are different from what one would expect from previous studies of nanowire growth and warrant some discussion. Typically, nanowire growth is controlled by the process of forming a nucleus of critical size at the growth front (*e.g.* the droplet–nanowire interface for vapour–liquid–solid growth, and the top surface for vapour–solid growth).^48–53^ Once the nucleus of critical size has formed, it propagates over the remainder of the growth front. In these growth scenarios, only one or a few steps exist at the growing surface on the top of the nanowire, which is confirmed by *in situ* TEM observation.^51–53^ This is because the energy required to form a stable nucleus is much higher than that to propagate over the growth front. For catalyst-free nanowire growth (*i.e.* vapour–solid growth), there is no preference for the location of the nucleation sites on the growth front, and nuclei were observed across the top surface.^52^ In comparison, we have observed many steps on the growth front surface and they are centred at the protuberance structures. These features suggest that the energy required to form stable nuclei was reduced to a level that is similar to that required for the propagation, and thus made the protuberance structures favourable nucleation sites.

**Fig. 5:**
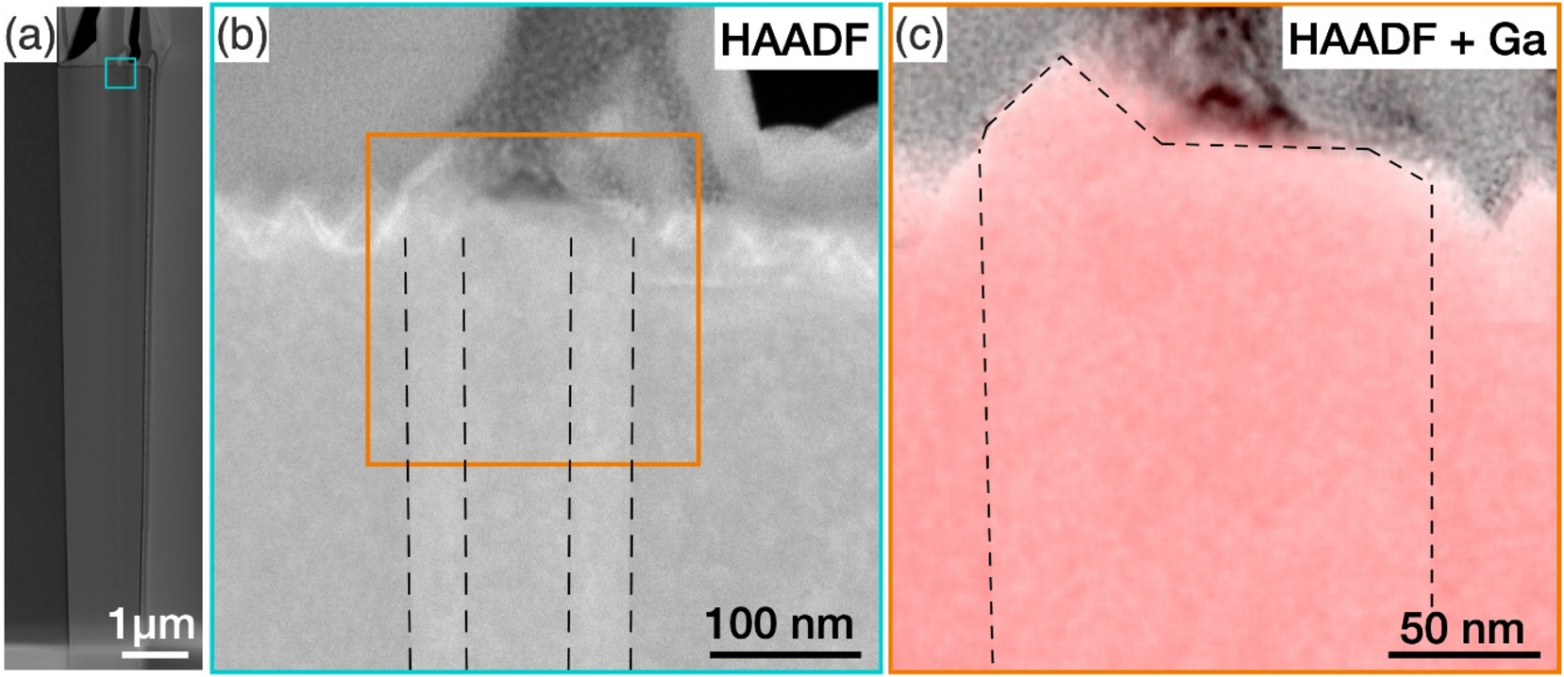
(a) HAADF TEM images of a tall GaN nanowire with a protuberance structure on the top surface. (b) HAADF image of the area marked in (a) where the protuberance structure was expected. (c) EDX map of the Ga signal superimposed on the HAADF image of the area highlighted in (b) with the protuberance structure and the hexagonal prism highlighted by dashed lines.

To investigate the structural properties of the protuberance structure, a cross-section of a tall GaN nanowire with such a structure was prepared by FIB. The nanowire was treated by KOH. Fig. 5(a) shows a high-angle annular dark field (HAADF) TEM image of the nanowire viewed along the 〈11̄00〉 zone axis. The GaN nanowire is slightly tapered with the diameter at the top of the nanowire being larger than the diameter at the bottom. The area where the protuberance structure was expected is highlighted by a square box, and a zoomed-in image of this region is shown in Fig. 5(b). A rough top surface was observed, which is caused by the KOH etching of the N-polar basal plane. The fade contrast reveals a hexagonal prism structure embedded in the N-polar nanowires along the whole observed length of more than 10 μm, highlighted by the dashed lines. In all examined nanowires with inversion domains, the inversion domain was present along the entire length. This suggests that the Ga-polar domain originates at the GaN/sapphire substrate, likely forming during the nucleation itself. The polarity of this prism was confirmed by CBED analysis to be Ga-polar, as shown in Fig. S8 of the ESI.†

Here, our findings are consistent with the work by Tessarek *et al.*, which showed that such Ga-polar domains originate at the GaN/sapphire interface. The authors speculated that the protuberance structures might have been formed as a result of faster growth rate of the Ga-polar (0001) plane compared to the surrounding N-polar (0001̄) plane at the nanowire growth front.^16^ To prove this suggestion, we have identified the tip of the Ga-domain by superimposing EDX maps of the Ga signal on the HAADF image in Fig. 5(c) (see Fig. S9† for the nitrogen elemental map). While a pyramid was observed at the top, the base of the pyramid covered only a small part of the Ga-polar domain, and a large part of the top surface of the Ga-polar domain remained flat. This indicates that the formation of the protuberance structure is not directly related with growth on the Ga-polar plane, which would otherwise have resulted in a pointing top surface comprised of inclined semipolar planes.^54^ Furthermore, we have shown in our nanowire length analysis in Section 3.2 that Ga-polar nanowires are significantly shorter than the N-polar nanowires, which clearly shows that Ga-polar growth is slower than N-polar growth. Combined with the observation of the concentric features at the protuberance structures (Fig. 4) and the slight tilt of the top surface (Fig. 3), we concluded that it is not the polarity of the Ga-polar domain below the protuberance structure, which promotes the growth of the nanowires with mixed polarity. Instead, the inversion domain boundaries provide energetically favourable nucleation sites at the growth front for both N-polar and Ga-polar planes.

### 3.4 Optical properties

**Fig. 6:**
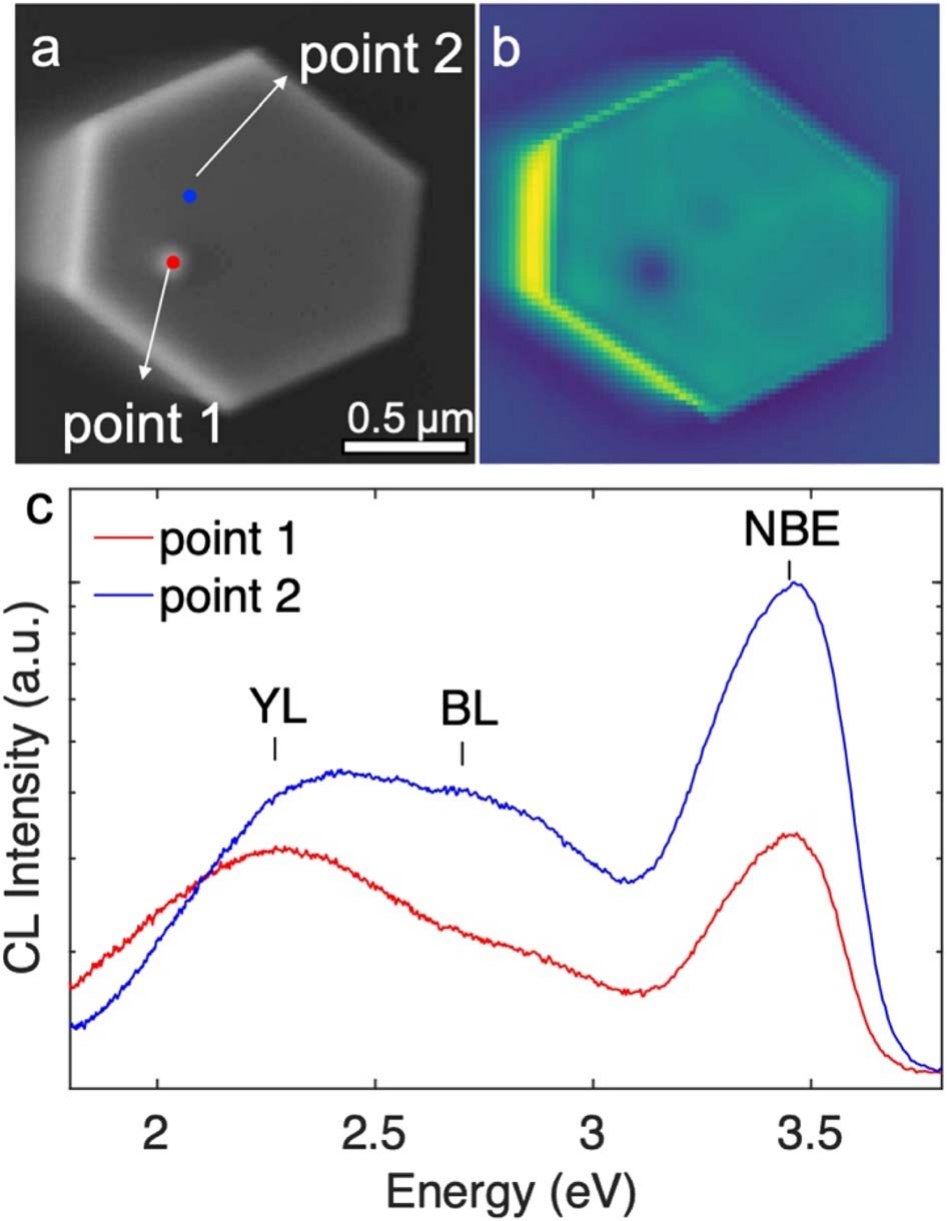
(a) SE image of the top surface of a nanowire. The blue and red circles indicate where the CL spectra in (c) were acquired. (b) The panchromatic map of the top surface. (c) CL spectra from the protuberance structure area (red) and the flat area outside of the protuberance structure (blue) with yellow-band (YL), blue-band (BL) and near-band-edge (NBE) emission labelled. The spectra were normalized to the near band edge emission from the flat area.

The impacts of the inversion domains and the protuberance structures on the optoelectronic properties were studied by room temperature cathodoluminescence (CL). The CL measurements were taken from the top surface of the same set of nanowires that was studied by AFM, and are shown in Fig. 6. The protuberance can be identified in the SE image (Fig. 6(a)) as a slightly brighter spot. The position of the protuberance correlates with a dark spot in the corresponding panchromatic CL image (Fig. 6(b)). Fig. 6(c) shows the CL spectra obtained from the region of the protuberance (point 1, red) and away from the protuberance (point 2, blue), as marked in Fig. 6(a). Both CL spectra show strong GaN near-band-edge (NBE) emission peaks at ∼3.45 eV and are accompanied by a broad yellow luminescence (YL) band near ∼2.2 eV and a blue luminescence (BL) band near ∼2.7 eV. The NBE emission and BL from the protuberance structure (point 1) is about a quarter and a third of the NBE emission and BL from the flat area (point 2), respectively. However, the YL from both areas is comparable. The contrast of NBE and BL between the N-polar region and the protuberance structure may be a result of enhanced radiative recombination efficiencies in the N-polar region (more Si incorporated) and/or increased rates of non-radiative recombination in the protuberance structure/Ga-polar domain.^55^ The electron depletion at the inversion domain boundary may play a role in decreasing the emission intensity at the protuberance structure as well. Nonetheless, the impact is limited to the protuberance structure area and does not extend to the surrounding areas, hence will not degrade the performance of devices whose functions are based on the sidewall facets.

## 4. Summary

In this work, we have systematically studied the initiation of the silane-assisted GaN nanowire growth on *c*-sapphire substrates. It is revealed that the sapphire surface undergoes a series of surface structure and composition change through the high temperature annealing under H_2_ ambient, the subsequent nitridation process and nucleation step. The nucleation step is critical for the subsequent silane-assisted GaN nanowire growth *via* transforming the AlN layer formed during the nitridation step to an AlGaN layer. Although the sapphire substrate was shown to be fully covered with a thin AlN layer during the nitridation step, both Ga- and N-polar GaN nanowires were grown. This suggests that Ga-polar domains could form as a result of the strain and surface tension relaxation of the surface nitride layer formed during the nitridation and nucleation steps.

The top surface of the GaN nanowires were studied by AFM, revealing dense ring-like features co-centred at the protuberance structures. While it was reported that the protuberance structures were formed due to faster growth rate of the Ga-domains, this conflicts with the fact that the growth rates of the pure Ga-polar GaN nanowires were much slower. Based on the observations, we propose that the inverse domain boundaries provide energetically favourable nucleation sites for the growth of both N-polar and Ga-polar planes, contributing to the fast growth rate of the silane-assisted GaN nanowire growth. Although CL studies shows a higher density of nonradiative recombination centres at the protuberance structures, their impact is highly localised and does not extend to the rest of the nanowires.

## Conflicts of interest

There is no conflict of interests.

## Supplementary Material

NA-005-D2NA00939K-s001
