# Supplementary material for: Complications in silane-assisted GaN nanowire growth
Source: Nanoscale Adv. 2023 Apr 20;5(9):2610–20. doi: 10.1039/d2na00939k (PMC10153487; doi:10.1039/d2na00939k)
Supplement: NA-005-D2NA00939K-s001 [file NA-005-D2NA00939K-s001.pdf]

Electronic Supplementary Material (ESI) for Nanoscale Advances.
This journal is © The Royal Society of Chemistry 2023

Supplementary Information

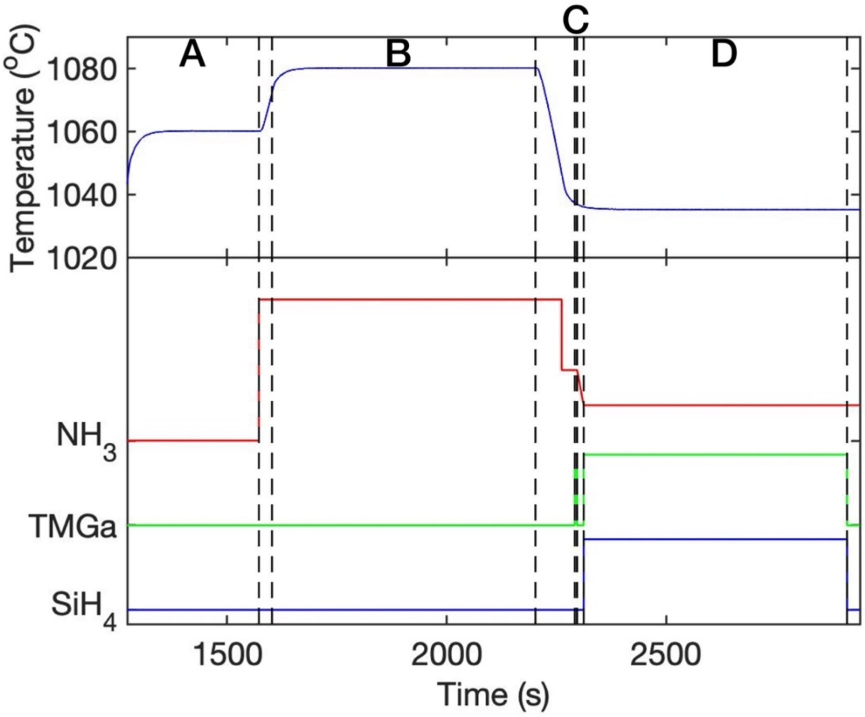

Figure S1. Wafer carrier temperature and precursor profiles for silane-assisted GaN nanowire growth. Step A is the high temperature annealing step at 1060℃ for 5 min in H_2. Temperature was then ramped up to 1080℃ for Step B the nitridation step for 10 min. 15L/min of NH_3 was used to achieve complete nitridation of the sapphire surface. After the nitridation step, the temperature and precursors (NH_3 and TMGa) were adjusted to prepare for the nucleation step. The nucleation step – Step C – only lasted for (0 – 5) s, after which the TMGa was switched off for 15 s while adjusting NH_3 for the next step. This 15 s is referred as the growth interruption. Step D is the silane-assisted growth where silane is introduced to promote GaN growth along the c-axis.

For Set A samples, the duration of Step C was set for 5 s and 0 s for the sample grown with and without the nucleation step, respectively.

For Set B samples, the process was terminated after Step A, Step B and Step C to study the substrate surface morphology and chemical compositions. These three samples were referred as Annealing, Nitridation and Nucleation respectively in Figure S2-S4 for the XPS studies. All XPS spectra were fitted with Pseudo-Voigt distributions.

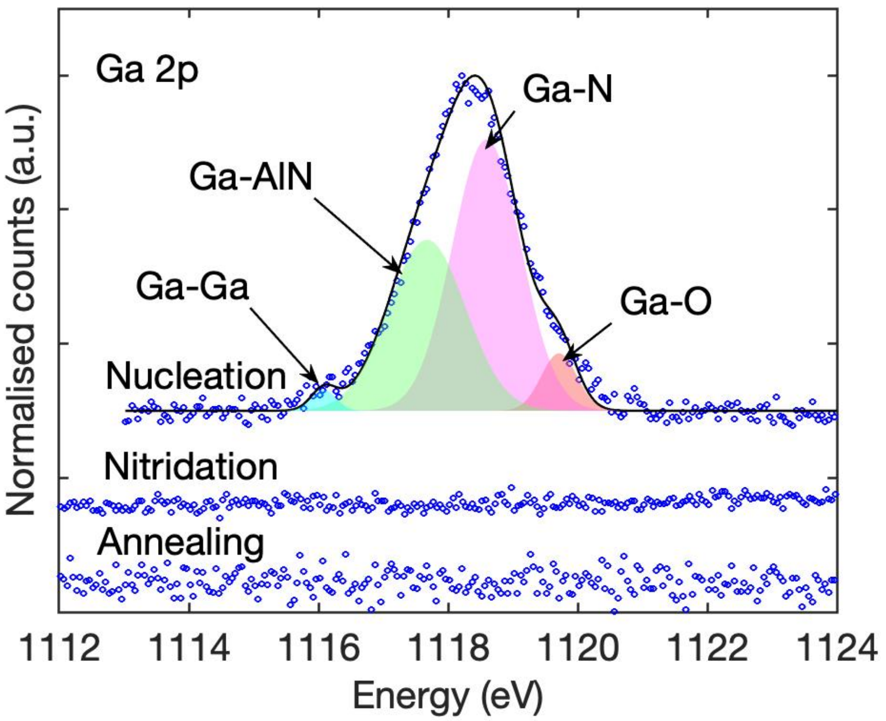

Figure S2. XPS spectra of Ga 2p measured from the samples with growth process terminated after the high temperature annealing step (Step A), nitridation step (Step B) and nucleation step (Step C), labelled as Annealing, Nitridation and Nucleation, respectively. Ga 2p peak was only observed from the Nucleation substrate.

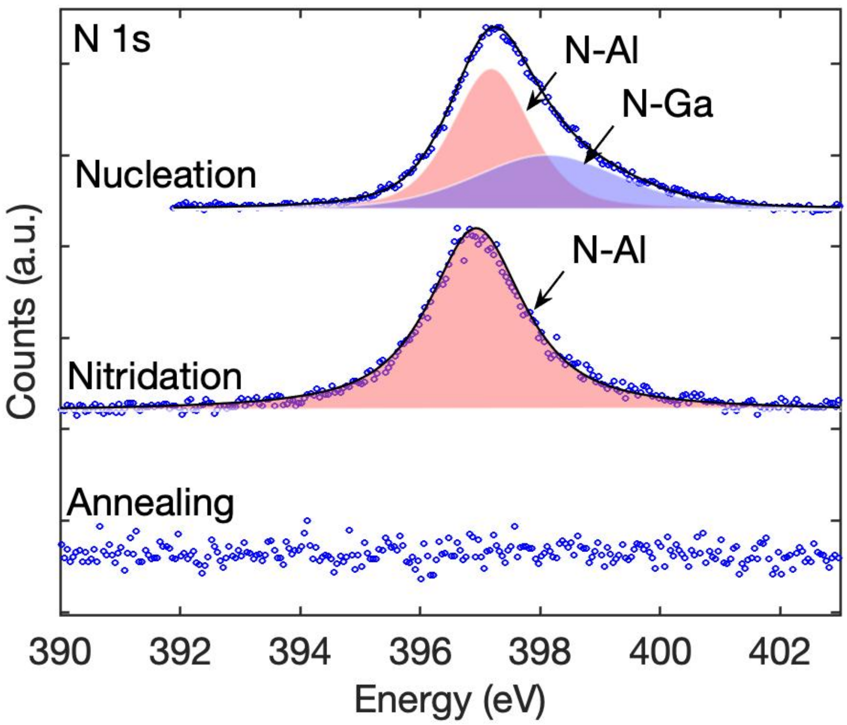

Figure S3. XPS spectra of N 1s measured from the samples with growth process terminated after the high temperature annealing step (Step A), nitridation step (Step B) and nucleation step (Step C), labelled as Annealing, Nitridation and Nucleation, respectively.

No N 1s peak was observed from the Annealing substrate. The N 1s peak from the Nitridation substrate was fitted with one Pseudo-Voigt distribution centred at 396.93 eV, attributing to the N-Al peak. The peak from the Nucleation substrate was best represented by fitting it with two Pseudo-Voigt distributions, centred at 397.18 eV and 398.12 eV, respectively. The 397.18 eV peak is from the N-Al bond[1,2] while the 398.12 eV is likely from the N-Ga bond.[3]

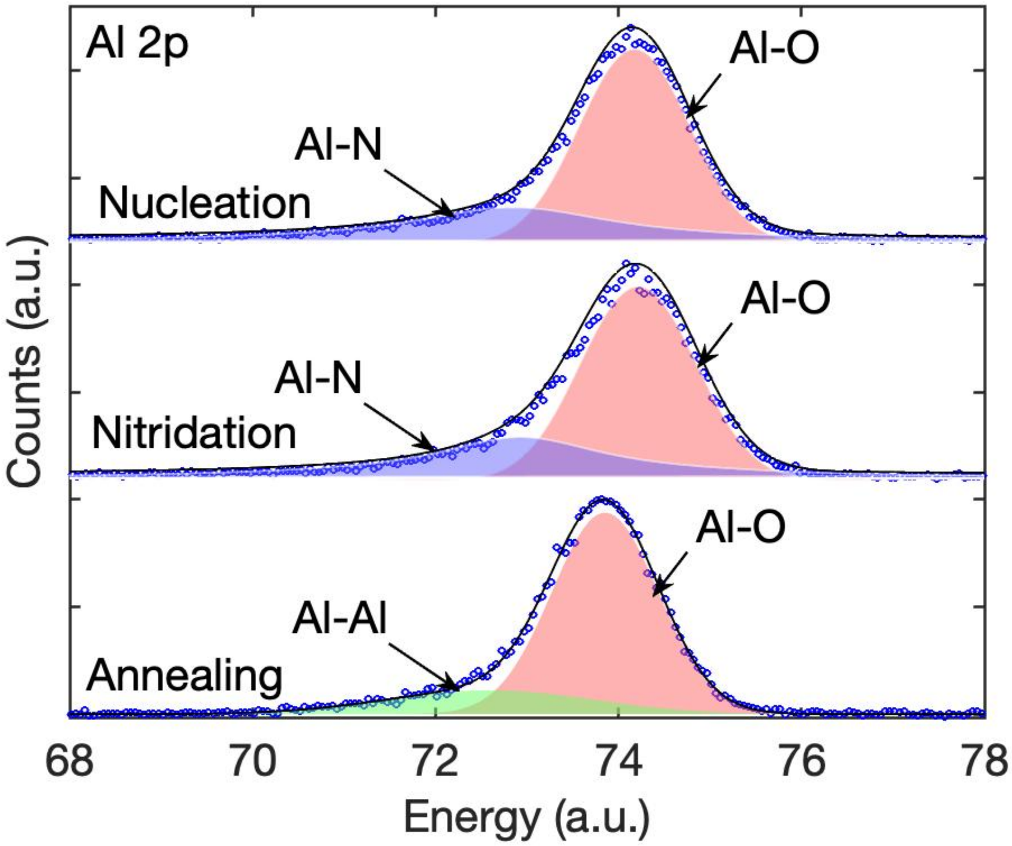

Figure S4. XPS spectra of Al 2p measured from the samples with growth process terminated after the high temperature annealing step (Step A), nitridation step (Step B) and nucleation step (Step C), labelled as Annealing, Nitridation and Nucleation, respectively.

All three spectra were dominated by the Al-O bonds, most likely from the sapphire substrate and the surface oxidation. The Al-O bond energy (73.89 eV) from the substrate after Annealing is slightly lower than that from the substrates after Nitridation (74.17 eV) and Nucleation (74.18 eV). This indicates that the Al-O bonds on the Nitridation and Nucleation substrates are more stable than that on the Annealing substrate. The lower Al-O bond energy for the Annealing substrate could be due to the contribution from the lattice deformation resulting from oxygen desorption.

The lower energy peak for the Annealing substrate was attributed to the Al-Al bond.[4] The lower peaks for the Nitridation and Nucleation substrates were likely from the Al-N bond.[5]

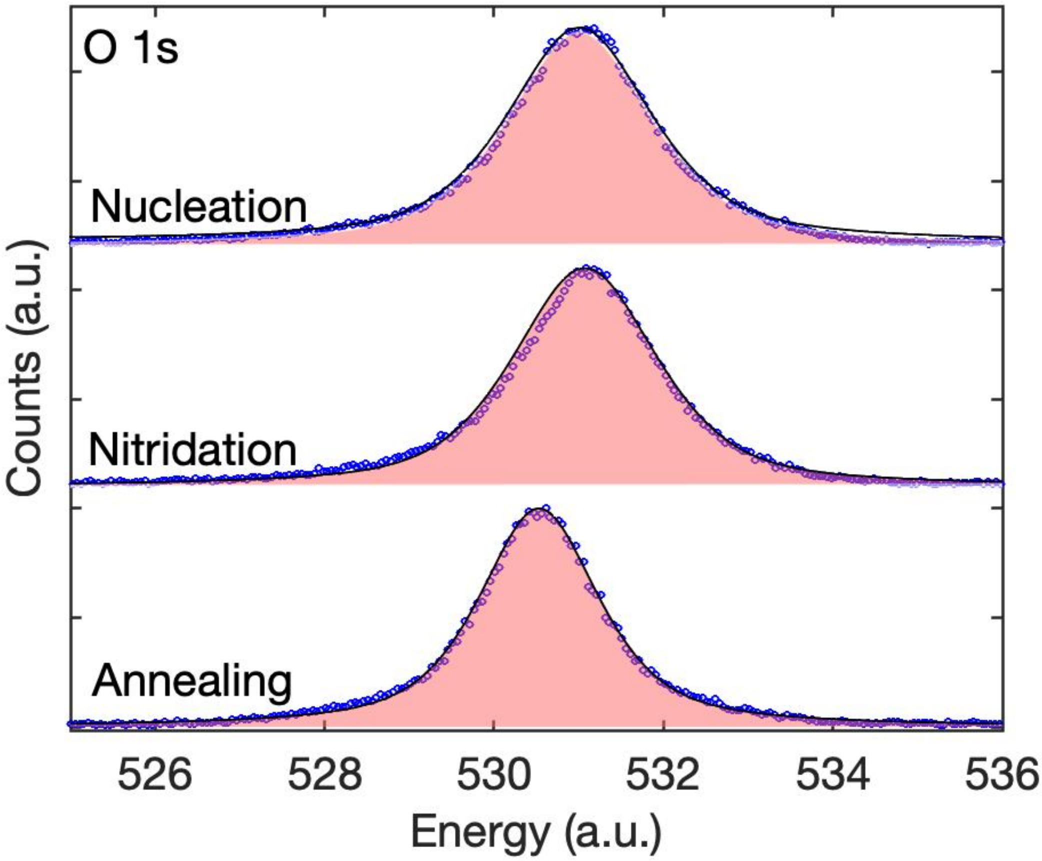

Figure S5. XPS spectra of O 1s measured from the samples with growth process terminated after the high temperature annealing step (Step A), nitridation step (Step B) and nucleation step (Step C), labelled as Annealing, Nitridation and Nucleation, respectively.

All three peaks were fitted with one Pseudo-Voigt peak. The peaks were centred at 530.53 eV, 531.07 eV and 531.01 eV for the Annealing, Nitridation and Nucleation substrates, respectively. The peaks from the Annealing and Nitridation substrates were attributed to the O-Al bond.[6,7] The energy of the O-Al bond from the Annealing substrate is shifted towards lower energy, consistent with the shift of Al-O bond in Figure S4. The peak from the Nucleation substrate may have contributions from both O-Al and O-Ga bonds.

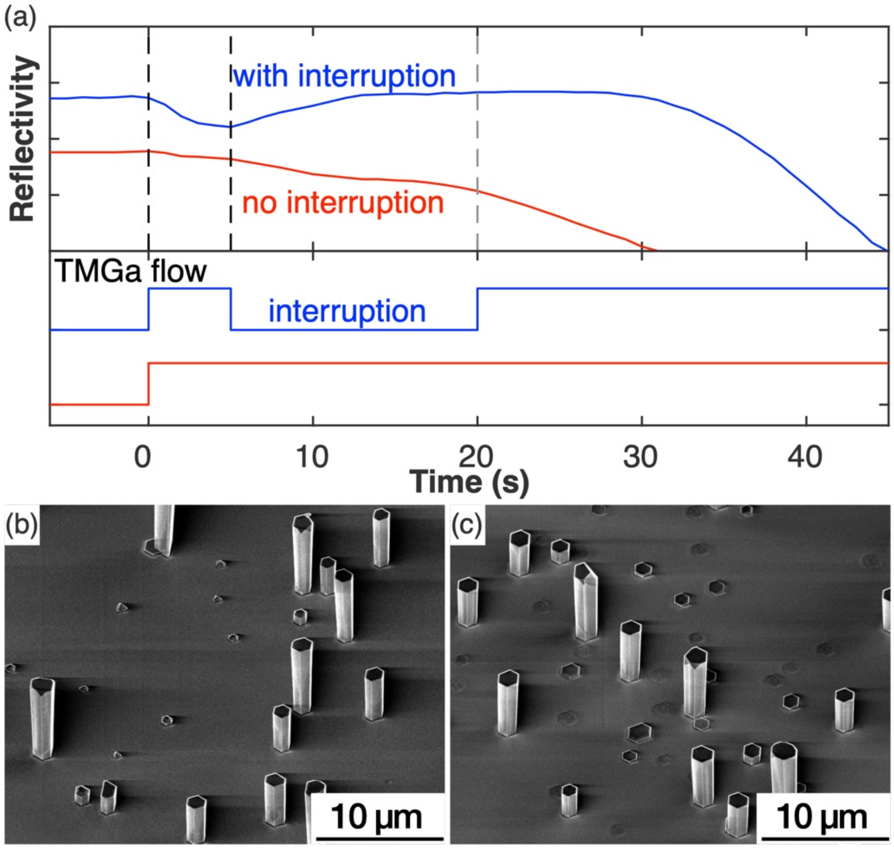

Figure S6. (a) In-situ reflectivity transients and the TMGa flow during the growth with (blue) and without (red) growth interruption. (b-c) SEM images of GaN nanowires grown with and without the 15 s growth interruption between the nucleation and silane-assisted growth steps.

The reflectivity of both samples started dropping immediately when TMGa was introduced into the reactor, likely due to the formation of 3D islands. For the sample without growth interruption, the reflectivity continued dropping as the growth parameters (silane introduction and temperature) were adjusted to promote nanowire growth, indicating the GaN islands remained present at the onset of Si-assisted growth in that case. When a growth interruption was employed, the reflectivity stopped dropping as soon as the TMGa flow stopped. Within 7 seconds of the growth interruption, the reflectivity recovered to its full value, suggesting a complete re-planarization of the substrate during the growth interruption.

Nanowires grew successfully in both samples, even after a growth interruption was employed to remove the island structures that formed in the nucleation step.

Although locally minor differences in the size and density of the nanowires can be observed across both samples, a statistical analysis across an area of about 1 cm^2 per sample has revealed no significant differences between them. The densities of the nanowires in both samples were measured to be ~ 5×10^5 cm^{-2}.

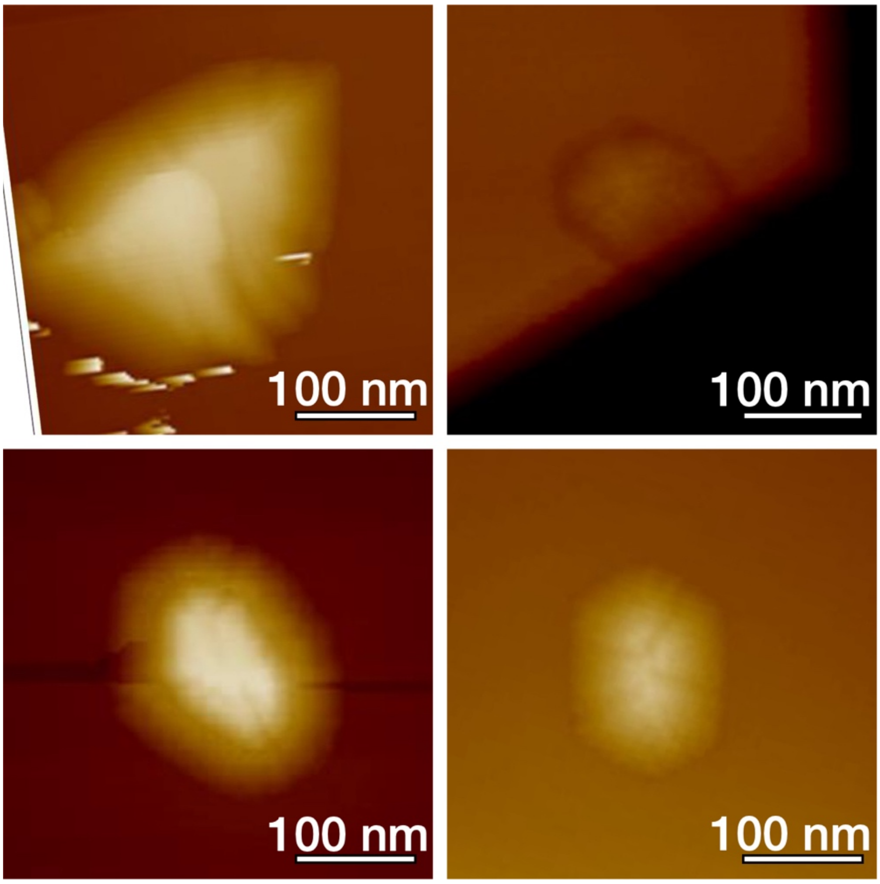

Figure S7. A collection of the topography of the protuberance structures from four different nanowires.

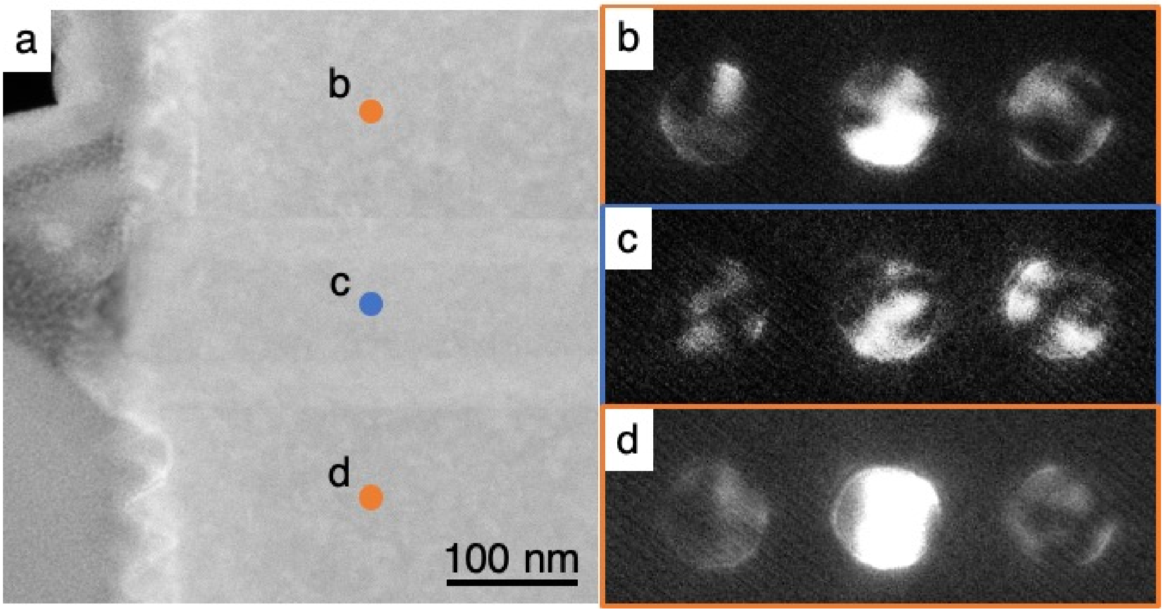

Figure S8. (a) High-angle annular dark field (HAADF) TEM image of the nanowire viewed along the $\langle 1\bar{1}00\rangle$ zone axis. (b-d) Convergent beam electron diffraction pattern (CBED) obtained along the $\langle 1\bar{1}00\rangle$ zone axis from the areas marked in (a). The patterns in (b) and (d) are similar to each other, yet distinctively different from the pattern in (c). This indicates the polarity of the domain where CBED pattern (c) was taken is different from the polarity of the areas where CBED pattern (b) and (d) were taken. The rough surface of (b) and (d) areas suggest N-polarity of these areas and we thus conclude the domain of c is Ga-polar.

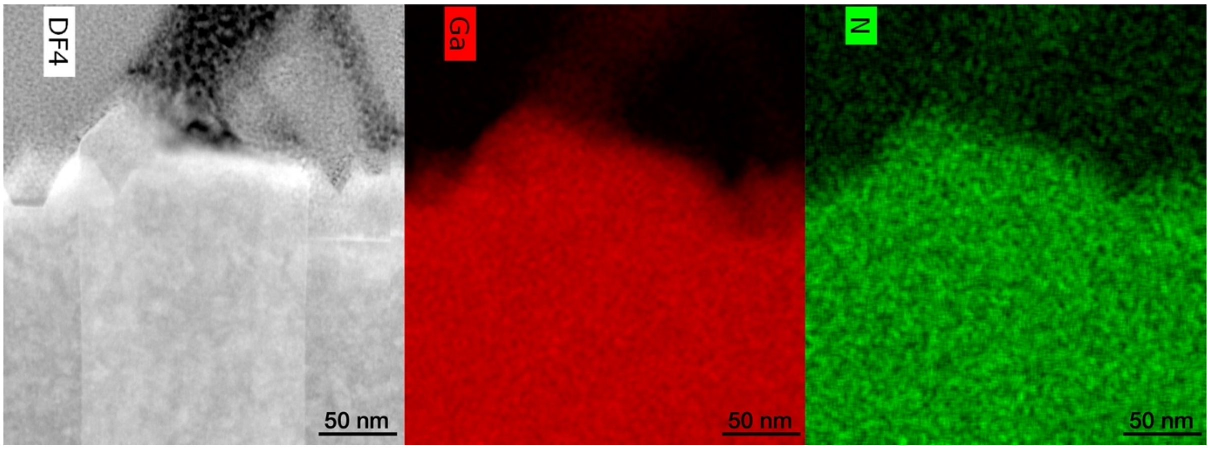

Figure S9. TEM image of the nanowire viewed along the $\langle 1\bar{1}00\rangle$ zone axis with the elementary maps of Ga and N of the same area, respectively.

References

[1] K. Yaddanapudi, S. Saha, S. Raghavan, K. Muraleedharan, and D. Banerjee, Cryst. Growth Des. **18**, 4978 (2018).

[2] P.W. Wang, J.C. Hsu, Y.H. Lin, and H.L. Chen, Appl. Surf. Sci. **256**, 4211 (2010).

[3] R. Jiang and X. Meng, J. Mater. Sci. Mater. Electron. **30**, 16266 (2019).

[4] T. Tago, N. Kataoka, H. Tanaka, K. Kinoshita, and S. Kishida, Procedia Eng. **216**, 175 (2017).

[5] T.T. Luong, B.T. Tran, Y.T. Ho, T.W. Wei, Y.H. Wu, T.C. Yen, L.L. Wei, J.S. Maa, and E.Y. Chang, Electron. Mater. Lett. **11**, 352 (2015).

[6] A.H. Alshehri, K. Mistry, V.H. Nguyen, K.H. Ibrahim, D. Muñoz-Rojas, M. Yavuz, and K.P. Musselman, Adv. Funct. Mater. **29**, (2019).

[7] G. Chavez-Esquivel, J.C. García-Martínez, J.A. de los Reyes, V.A. Suárez-Toriello, M.A. Vera-Ramírez, and L. Huerta, Mater. Res. Express **6**, 105201 (2019).
